# Supplementary material for: Integrated Transcriptomics and Metabolomics Provide Insight into Degeneration-Related Molecular Mechanisms of Morchella importuna During Repeated Subculturing
Source: J Fungi (Basel). 2025 May 30;11(6):420. doi: 10.3390/jof11060420 (PMC12194730; doi:10.3390/jof11060420)
Supplement: Supplementary file 1 [file jof-11-00420-s001.zip › Supplementary figures.pdf]

Supplemental figures

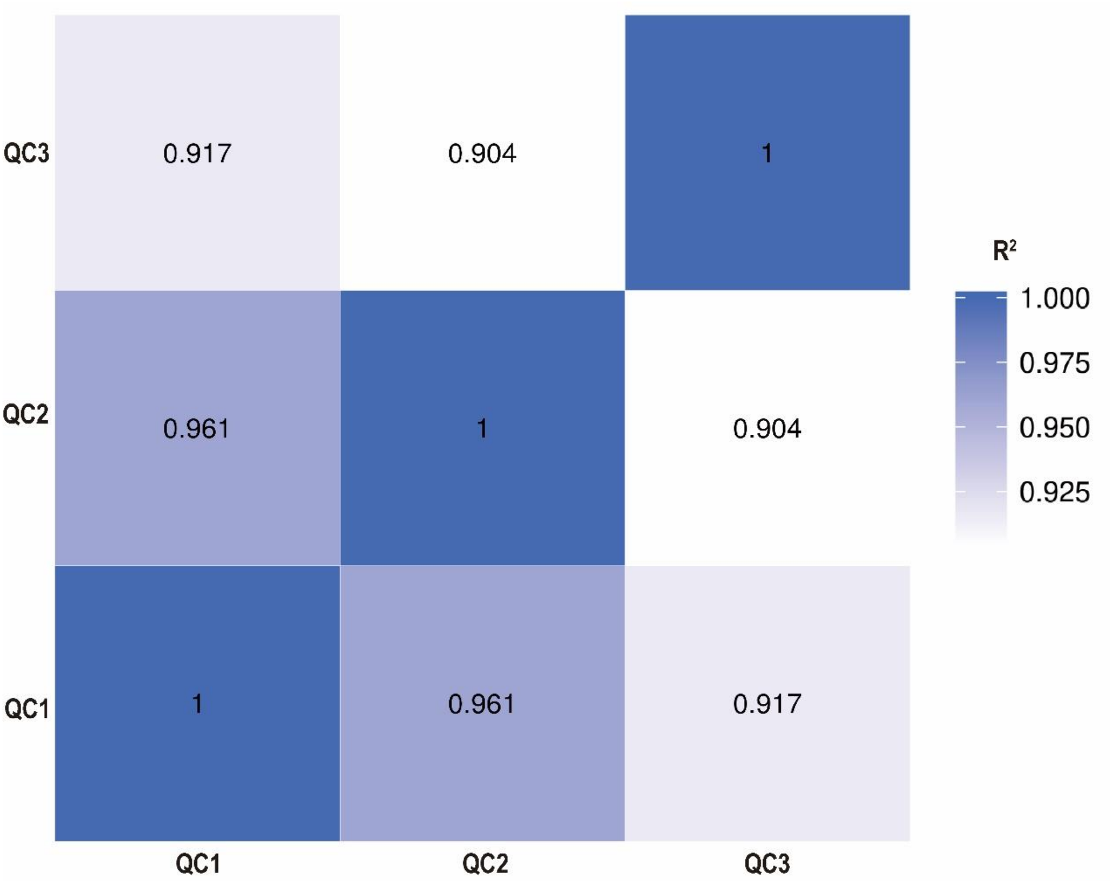

Figure S1. Pearson correlation analysis between QC samples.

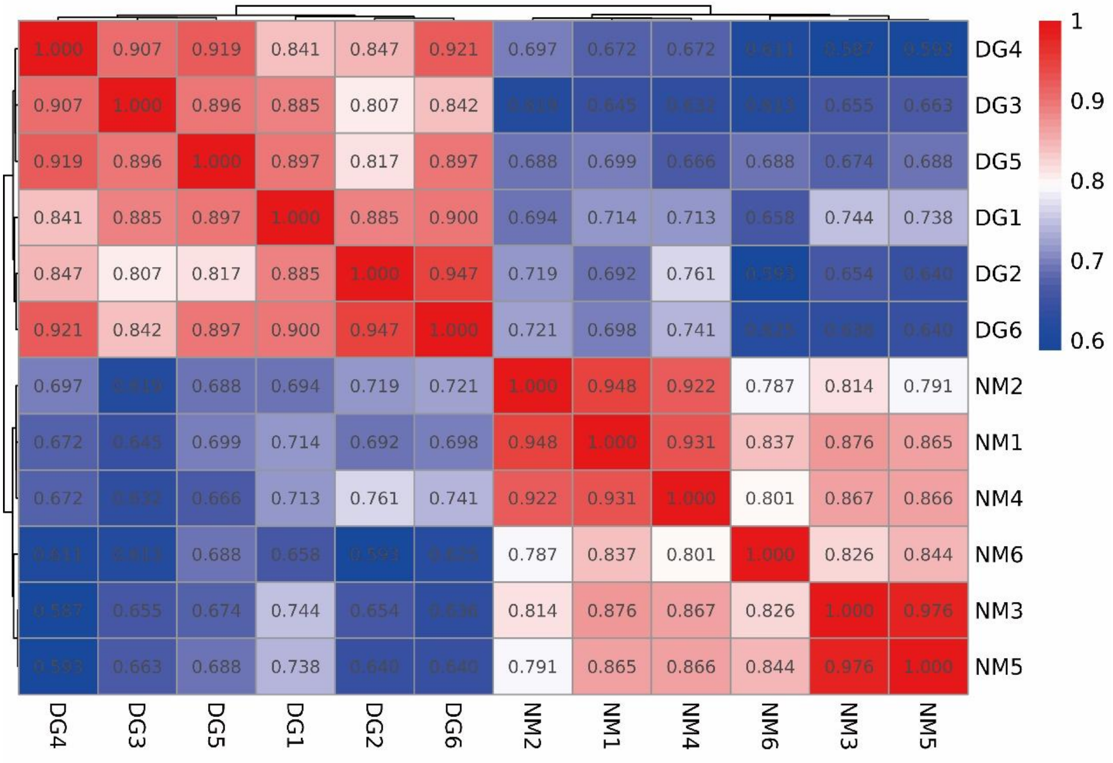

Figure S2. Pearson correlation analysis between all metabolome samples.

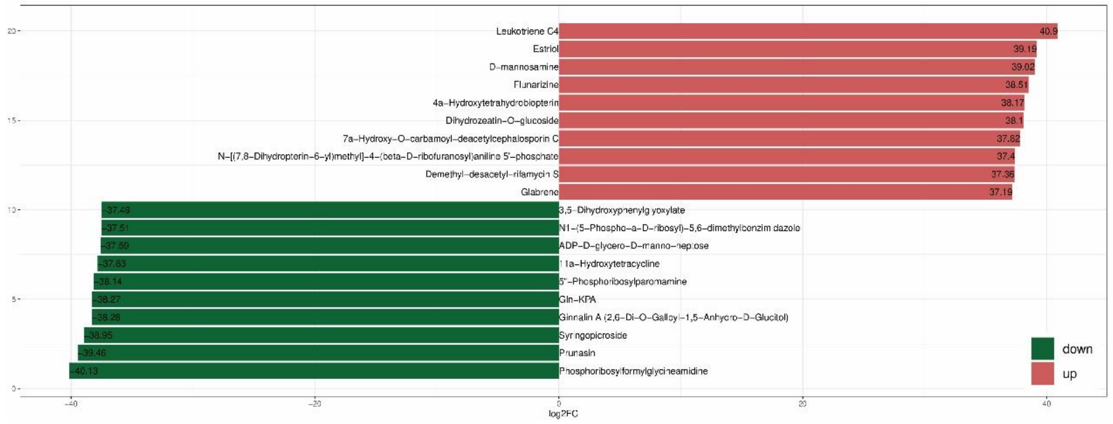

Figure S3. The top 20 up- and downregulated DEMs between NM and DG groups.

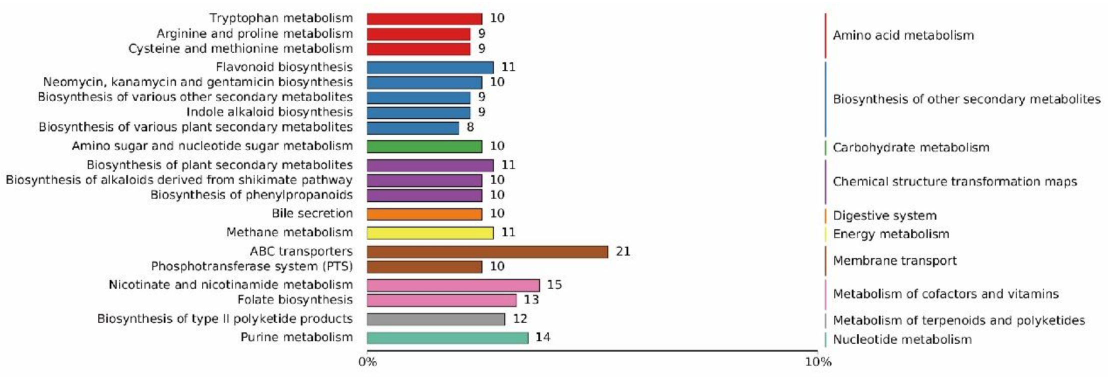

Figure S4. The top 20 KEGG pathways with the most DEMs.

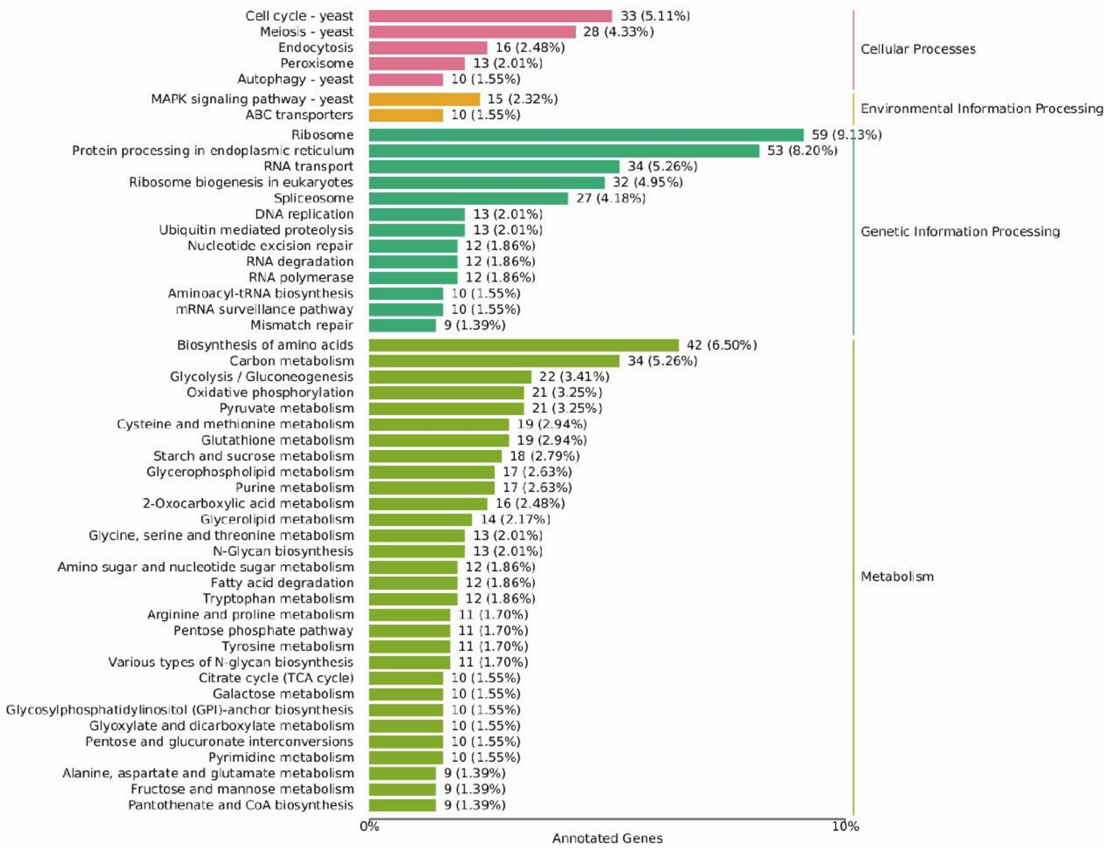

Figure S5. KEGG enrichment analysis of DEGs.

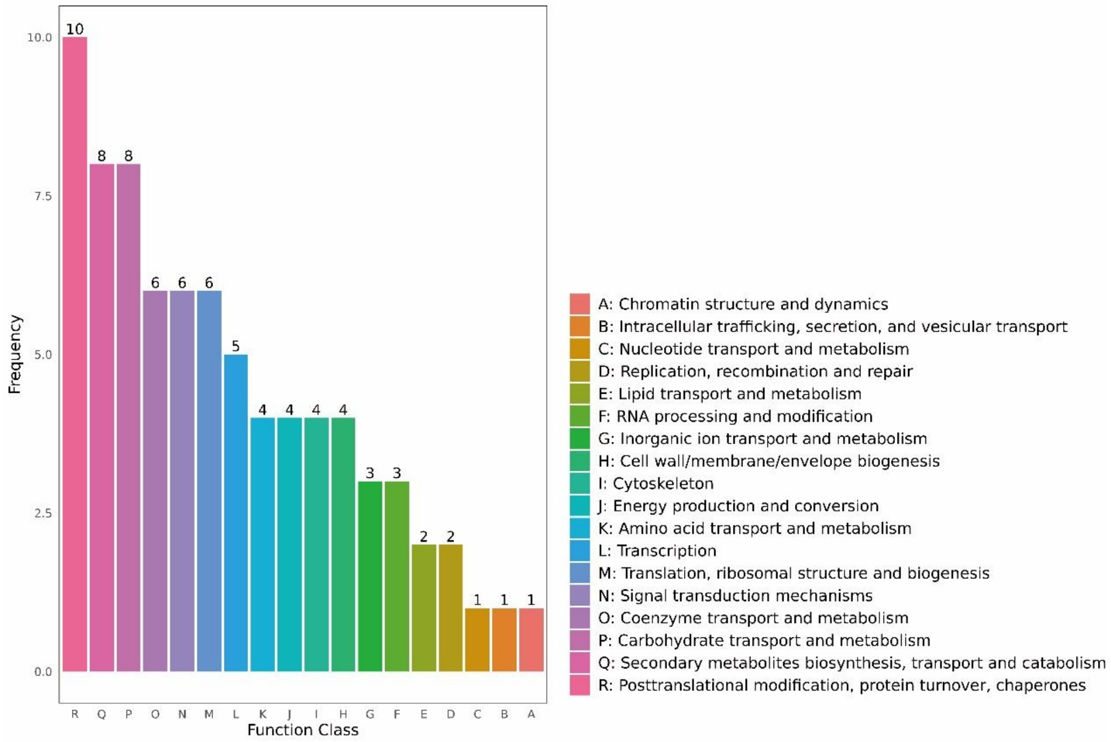

Figure S6. eggNOG function classification of DEGs.

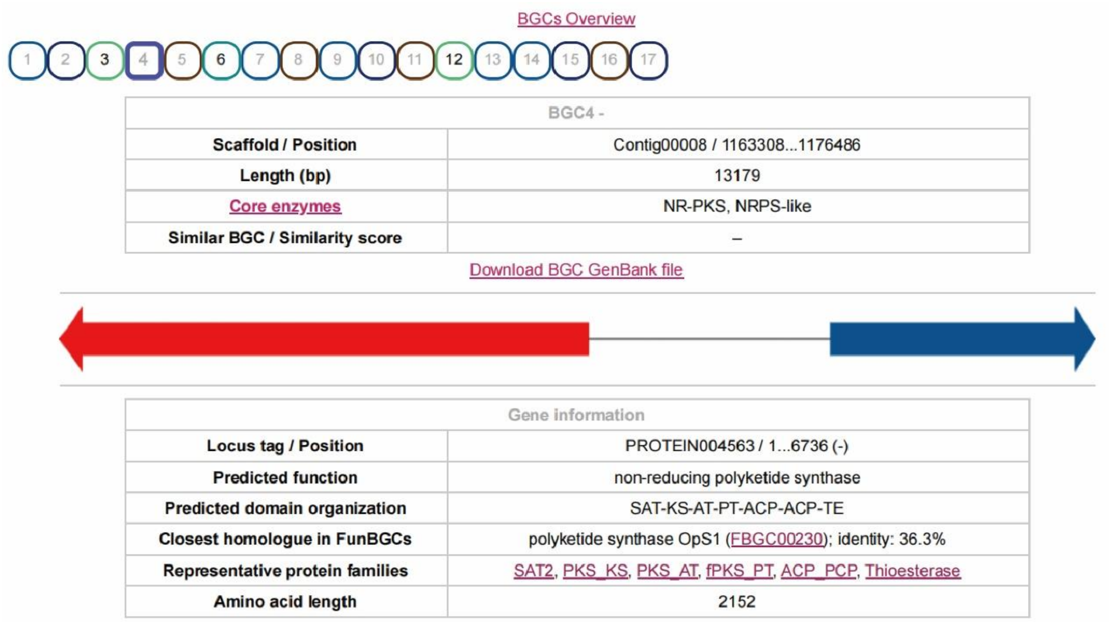
BGCs Overview

1 2 3 4 5 6 7 8 9 10 11 12 13 14 15 16 17

BGC4 -	
**Scaffold / Position**	Contig00008 / 1163308...1176486
**Length (bp)**	13179
**Core enzymes**	NR-PKS, NRPS-like
**Similar BGC / Similarity score**	–

Download BGC GenBank file

Gene information	
**Locus tag / Position**	PROTEIN004563 / 1...6736 (-)
**Predicted function**	non-reducing polyketide synthase
**Predicted domain organization**	SAT-KS-AT-PT-ACP-ACP-TE
**Closest homologue in FunBGCs**	polyketide synthase OpS1 (FBGC00230); identity: 36.3%
**Representative protein families**	SAT2, PKS_KS, PKS_AT, fPKS_PT, ACP_PCP, Thioesterase
**Amino acid length**	2152

Figure S7. The discovery of novel fungal BGCs from the genome of *Morchella importuna* using the FunBGCeX tool.

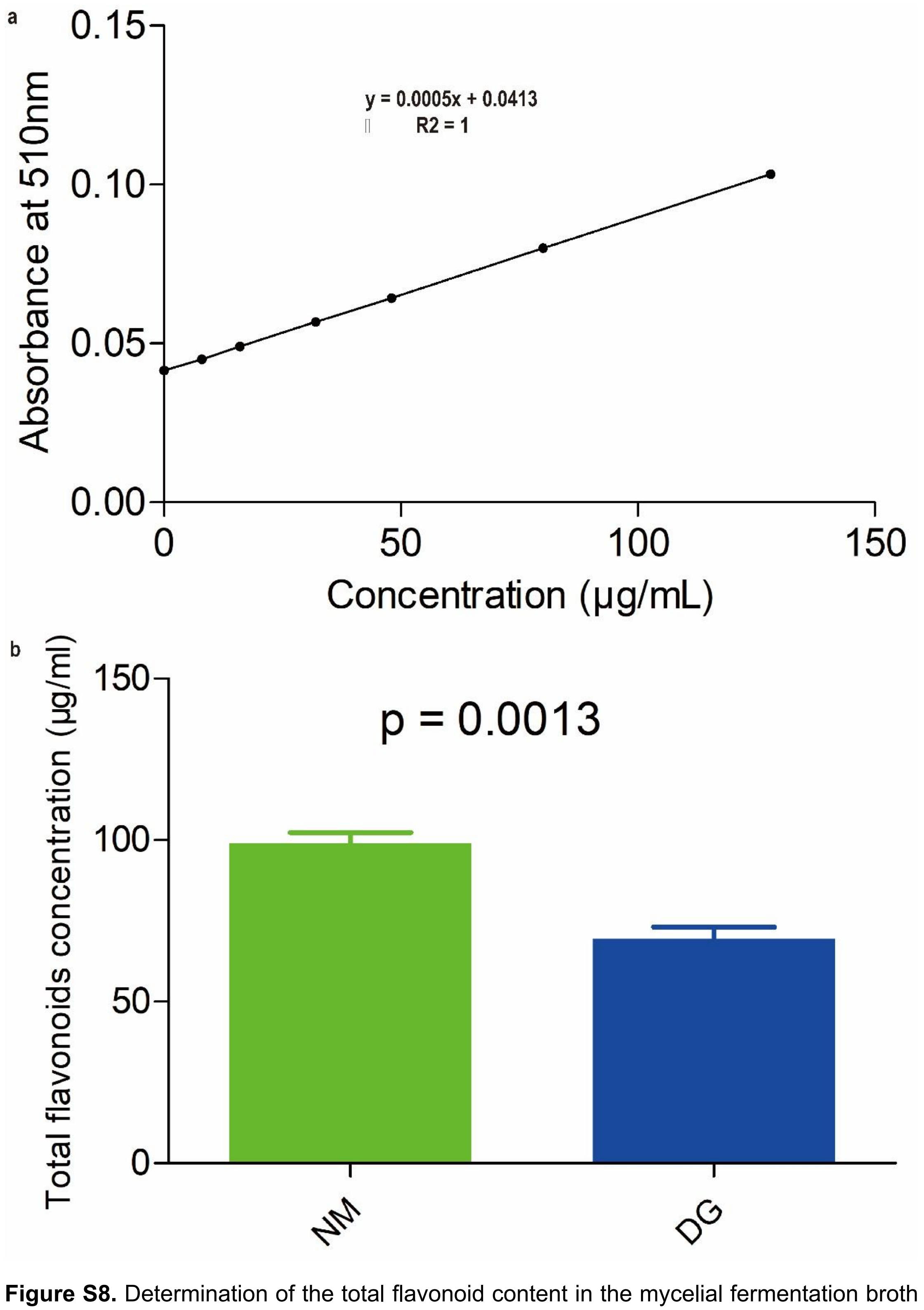

Figure S8. Determination of the total flavonoid content in the mycelial fermentation broth of the normal and degenerated strains. Standard curve for total flavonoids determination (**a**); the total flavonoid content of samples of NM and DG groups (**b**), *t* tests were used to evaluate the significant differences, each value is presented as mean ± SD.

.
